# A Health System Framework for Addressing Structural Racism: Mass General Brigham's United Against Racism Initiative

**DOI:** 10.1089/heq.2023.0077

**Published:** 2023-09-13

**Authors:** Allison S. Bryant, Julia A. Healey, Sarah Wilkie, Carla Carten, Thomas D. Sequist, Elsie M. Taveras

**Affiliations:** ^1^ Mass General Brigham Health System, Somerville, Massachusetts, USA.; ^2^ Massachusetts General Hospital, Boston, Massachusetts, USA.; ^3^ Brigham and Women's Hospital, Boston, Massachusetts, USA.

**Keywords:** structural racism, antiracism, health system, health equity, social risk mitigation

## Abstract

The legacy of racism and structural inequality has taken a heavy toll on the health care system and the health outcomes of patients and members of community catchment areas. To achieve optimal health outcomes for all, health systems will need to enact structural change that is meaningful, measurable, and rooted in evidence. We describe an antiracism campaign organized into three pillars of focus (*Leadership/Employees/Culture, Patient Care Equity,* and *Community Health and Policy Advocacy*) and implemented across Mass General Brigham, a large integrated health system in the northeast of the United States. Our study ranges from the foundational to the aspirational and examples of data-driven areas of focus, programs (e.g., staff education, social risk mitigation, and new models of clinical service), and metrics developed for the health care workforce, patients, and surrounding communities are presented.

## Introduction

Racism is inextricably linked to the U.S. health care system and contributes to profound and well-documented inequities in health outcomes.^1–3^ Structural racism, the ways in which societal and institutional policies and practices foster and reinforce racial discrimination, has led to the inequitable distribution of health-promoting resources between populations, including access to health care services and social determinants of health (SDOH).^4,5^ Persistent racial and ethnic health inequities and the heightened national attention to racism in 2020 in the United States highlighted the need to re-evaluate health policies and practices rooted in structural racism. Health care institutions have a responsibility to address structural racism and can play an integral role in promoting health equity.

As the largest health care system in New England, employing >80,000 employees and serving >2 million patients annually, Mass General Brigham has long recognized the barriers to equitable outcomes that exist within our own organization and the communities we serve. Made up of 12 hospitals and affiliates across a multitude of care settings, from academic medical centers to community hospitals and health centers, we provide the range of primary to specialty quaternary care and serve people from diverse communities, many of which have been historically marginalized.

Given our opportunity to embed structures and processes to achieve equity in health, we are directing our resources to implement wide-scale change to eliminate structural racism across our system. Our goal is to enact system change and to create a culture of workforce inclusion, clinical and community health equity, where every person has the opportunity to attain their full health potential, and no one is disadvantaged due to any socially determined circumstances.^6^

To eliminate the impact racism has on our patients, communities, and workforce, we are working to dismantle the systems, policies, and actions within and outside our walls that create and maintain inequities. In October 2020, we launched a system-wide initiative to become an antiracist health care organization, United Against Racism (UAR).^7^ UAR is a strategic commitment to bring our system to a higher level of leadership and accountability in dismantling structural racism. Our UAR efforts are organized by interconnected workstreams under three pillars: (1) Leadership, Employees, and Culture; (2) Patient Care; and (3) Community Health and Policy Advocacy.

## Background

UAR is a product of collaboration between Mass General Brigham's leadership, employees, and the communities we serve. We first explored and documented racial and ethnic disparities within our patient outcomes and community health programs and identified contributing practices and policies. Members of the Mass General Brigham community have long been engaged in health equity improvement work, sometimes in silos; UAR provides greater resources and an explicit coordinated approach to address inequities in clinical and community health across our system. Though this plan addresses structural contributors across our enterprise, it also allows for adaptation to the local needs of facilities and communities within our system.

**FIG. 1.**
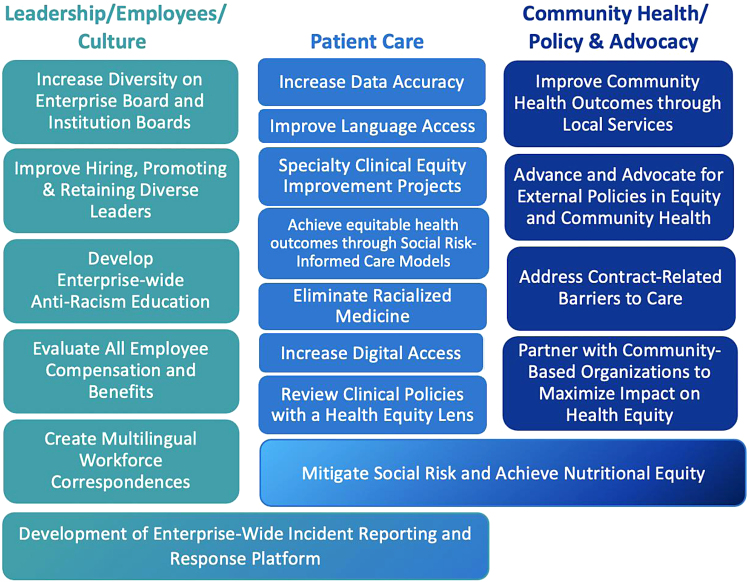
Mass General Brigham United Against Racism pillars and workstreams.

Work to transform our workforce into an antiracist community falls under the purview of Diversity, Equity, and Inclusion and Human Resources professionals under *Leadership/Employees/Culture, while the* overarching goals of the *Patient Care Equity,* and *Community Health and Policy Advocacy* pillars (Fig. 1) are to improve health and equity among our patients and communities we serve.

Here we describe selected work under each pillar, including our methods, measurable goals to monitor progress (Table 1), and outcomes where available.

**Table 1.** Selected Mass General Brigham United Against Racism Workstreams, Goals, and Planned Metrics

Workstream	Goals	Planned metrics
Patient Care Equity		
Patient race, ethnicity, and language data accuracy	Achieve <5% missing self-reported patient race, ethnicity, ethnic background, written, and spoken language demographic data	Percentage missing data in each category (i.e., response=“Unknown”; “Declined” response counted as nonmissing)
Digital access	Enable equitable access to patient portal and clinical digital tools	Enrollment in patient portal by patient race, ethnicity, and language Engagement with DAC corps
Language access	Provide written and verbal communication in patient preferred language	Percentage of key patient-facing documents translated into top six languages across system Documentation of use of trained medical interpreter or qualified bilingual staff for patients reporting need
Achieving equitable health outcomes through social risk-informed care models	Improve hypertension control in Black and Hispanic patients by 5%; increase participation in bridge clinics in Black, Hispanic, and non-English-speaking patients by 5%	Hypertension control by race/ethnicity within participating primary care practices Number of visits to traditional and mobile bridge clinics by race/ethnicity and language
Mitigating social risk	Assess for patients' unmet health-related social needs and provide resources and referral	Percentage eligible patients screened Response rates for positive screens
Specialty clinical equity improvement projects	Design and execute equity improvement work to close inequities specific to each specialty department	Process and outcomes metrics unique to each project
Eliminating racialized medicine	Eliminate the use of clinical calculators and algorithms that use individual race or ethnicity as inputs	Number of reviewed, revised, and substituted calculators and algorithms Downstream consequences of changes
Leadership/Employees/Culture		
Develop enterprise-wide antiracism education	Engage all Mass General Brigham employees in series of antiracism training	Percentage of employed staff participating in each training
Instances of racism reporting	Develop and socialize trusted means of reporting and reconciling instances of racism and discrimination within our medical system community	Number of reports filed and completed Number of managers and leaders who have completed Upstander training Staff and patient experience survey responses
Community Health and Policy Advocacy		
Community-based clinical equity programs	Launch community-embedded mobile service line dedicated to care of hypertension and substance use disorder, paired with social risk mitigation	Patient volume in mobile setting
External policy and advocacy	Develop policy agenda to support and advocate for state and federal policy to facilitate health equity	Annual revision of policy agenda Number of completed Requests for Information from state and federal agencies

DAC, Digital Access Coordinator.

## UAR Workstreams

### Patient care equity

#### Patient race, ethnicity, and language data accuracy

Collecting accurate race, ethnicity, and language (REaL) data for the patients we serve is critical to identify and target for reduction inequities in health care programs and services: what cannot be measured cannot be improved.^8,9^ Under the *REaL Data Accuracy* workstream, we are achieving greater accuracy of self-reported REaL data capture within our electronic medical records. We began this study within our adult primary care population where at baseline in 2020, documentation of race was missing for 5% of the adult primary care population, 17% missing for ethnicity, and 4% missing for language. The objective for this workstream is to reach and maintain <5% missingness for each category.

To achieve our goal, we are leveraging centralized registration capacity to enact standardized and informed methods to collect REaL patient data across all sites within our network. Recognizing the critical role of the data collector, we have developed training materials for registrars to share the value of accurately capturing REaL data. Registrar staff were provided with tools to communicate the rationale to patients for whom mistrust and misinformation might guide their inclination to participate.

Given our consideration of self-report as the gold standard for this data collection, this study cannot be done well without patient engagement. We thus conducted direct patient outreach to collect up-to-date REaL data through paper and electronic patient surveys, reaching out to >1 million patients across our system. Patients are also prompted to review and update their demographic information in our electronic patient portal at the time of check in for ambulatory visits. At present, we achieved rates of missing race and language of <5%, but 10% of patients remain without documentation of ethnicity (i.e., Hispanic or not Hispanic).

In addition, we have created patient-facing communication materials for use at clinical sites that describe the importance of collecting REaL data and that share actionable steps patients can take to update their information. Building on our strategies for collection of REaL data, we are now moving toward improvement in collection of self-reported sexual orientation, gender identity, disability identity, and need for accommodations in the health care setting.

#### Digital access

There exists a “digital divide” that separates those with greater access and skills to use technology and the internet in the United States from those with fewer technological resources and literacy.^10^ The coronavirus disease (COVID-19) pandemic highlighted existing racial and ethnic inequities in access to now-essential virtual health care,^11^ and also solidified virtual care as a standard for the future. Within Mass General Brigham, we documented inequities between races and ethnicities in access to and use of our online patient portal, *Patient Gateway*, which is the most streamlined way to engage in virtual care, participate in self-monitoring, and leverage communications. Under the *Digital Access* workstream, we aim to increase *Patient Gateway* enrollment overall, and for Black and Latinx patients by 15% over baseline in 2 years, targets that have been met and exceeded.

This workstream addresses barriers in access to internet-enabled devices required to participate in virtual care, as well as improving digital literacy skills. To increase equity in technology access, we have 2000 internet-enabled tablets available for patients without ready access to such devices and with specific clinical needs (e.g., chronic conditions such as hypertension). We also established a new workforce of digital access coordinators (DACs). DACs represent multilingual corps, many of whom hail from the same communities as the patients we serve, which are available to teach patients the technical features of the devices and applications needed to manage their health.

In addition, DACs are available to assist patients in enrollment and effective use of *Patient Gateway*. Some DACs are embedded in our clinics located in historically marginalized communities, whereas others work centrally with similar content focus, completing referrals across the health system. The introduction of these programs has been associated with reductions in racial and ethnic gaps in *Patient Gateway* enrollment.

#### Language access

Individuals with limited English proficiency (LEP), who have difficulty speaking, reading, writing, or understanding English, experience barriers accessing quality health care in the United States.^12^ This contributes to worse health outcomes and greater social needs among LEP racial and ethnic populations compared with English-proficient patients.^13–15^ Similarly to inequities in digital access, racial and ethnic disparities in access to language-concordant clinical information and care have been exacerbated by the COVID-19 pandemic.^16,17^

Among our patient population at Mass General Brigham, ∼5% are LEP, with higher proportions of non-English speakers cared for in our academic medical centers, where the prevalence is as high as 10%. The goal of the *Language Access* workstream is to improve access and quality of our care to patients for whom English is not their primary language, through expanded written translation, then ultimately improved oral interpretation and access to qualified bilingual staff.

We created a central operation to translate written patient-facing materials in the top six non-English languages spoken by our patients across our system: Arabic, Portuguese, Haitian Creole, Russian, Spanish, and Traditional Chinese. Teams are staffed to provide quality-checked translations of clinical and administrative patient-level documents that are centralized across the health system, including ∼1000 patient questionnaires, 810 patient-facing communication materials, and >1000 COVID-19–related documents. As above, translating our online patient portal was also an important step toward equity.

Previously *Patient Gateway* was only available in English and Spanish; now all of the static content is translated into the six top patient languages. This widespread translation endeavor was, to best of our knowledge, the first of its kind for an Epic Systems client. The increased access to the patient portal afforded by translation has led to increased demand for engagement with dynamic tools on the platform.

#### Achieving equitable health outcomes through social risk-informed care models

We recognize that inequities in health outcomes and variation in access and quality of health care exist between populations in essentially every field of medicine and health condition. To maximize the impact of our efforts, we determined those acute and chronic conditions that are especially large contributors to excess deaths among Black individuals in our communities. This led us to center our work on inequities in hypertension^18^ and substance use disorders^19^ as the main foci of our UAR clinical efforts. To this end, we have identified 29 primary care practices that in 2020 had high burdens of COVID-19 infection and other chronic conditions.

**FIG. 2.**
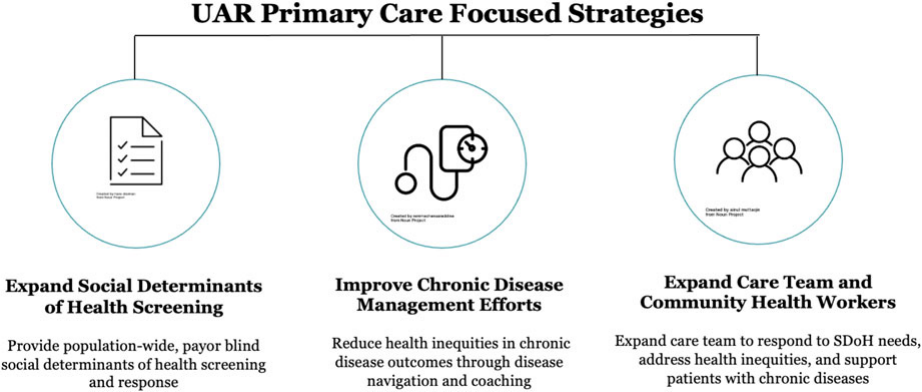
Social risk informed hypertension care planning.

These targeted practices have received dedicated resources to conduct surveillance for patients with uncontrolled hypertension. These patients, as with all others in the practice, are screened for SDOH and are offered partnership with a community health worker (CHW) who assists with finding resources to match the need (e.g., food insecurity, transportation challenges, and help with utilities) while also providing a bridge between the patient and clinicians; the CHWs facilitate remote blood pressure monitoring and management in this model (Fig. 2). Goals for this program include improvement in hypertension control for Black and Hispanic patients by 5% in 1 year.

To address substantial disparities in overdose deaths among Black individuals,^20^ we are building capacity and intent to enroll greater number of Black, Hispanic, and non-English-speaking patients into our bridge clinics, offering evidence-based low barrier care to those with substance use disorder. Care is provided in traditional brick and mortar health care sites in four of our communities, as well as in mobile care settings, and includes screening for and mitigating unmet social need.

In crafting our roadmap to achieve health equity, we will also develop focus areas in maternal health and cancer screening and treatment given their contributions to excess mortality among populations in our catchment areas.

#### Mitigating social risk

More than 80% of the variation in health outcomes is related to factors other than medical care, such as nutrition, housing, transportation, employment, and education.^21^ Structural racism has resulted in inequitable conditions in which people are born, grow, live, work, and age, known as the SDOH.^4^ Screening patients in clinical settings for SDOH risk factors helps to document inequities in social need and to connect patients with needed resources.^22^ Under our *Mitigating Social Risk* workstream, we are expanding our SDOH screening among many of our primary care patients and working to connect those with unmet social needs to appropriate resources.

Beginning in 2018, as part of a contractual obligation, primary care patients enrolled in the Mass General Brigham Accountable Care Organization (ACO) were screened annually for SDOH with referrals made to social service organizations or written resource information given. With the advent of our UAR campaign, SDOH screening and response were introduced in 16 adult and 6 pediatric primary care practices in a payer-blind manner. In selecting the practices for early inclusion, we focused specifically on communities with known health inequities, such as in chronic disease or COVID-19 outcomes.

As part of our efforts, we expanded our teams' capacity to respond to positive screenings by augmenting our corps of CHWs to connect patients who screen positive to resources. Leveraging CHWs' skills and connections to the community is integral to this work to enhance the prevention and early detection of some of the most persistent diseases and conditions facing historically marginalized communities.

#### Specialty clinical equity improvement projects

Beyond outcomes and care processes associated with adult primary care, inequities in other care spaces remain prominent. As examples, Black and Hispanic birthing people are more likely to undergo cesarean delivery,^23^ and less likely to engage in advance care planning and palliative care. These inequities are seen both nationally and within our care system. Under the *Specialty Department Equity Goals* workstream, we are measuring, monitoring, and addressing structural racism in clinical settings more broadly.

**FIG. 3.**
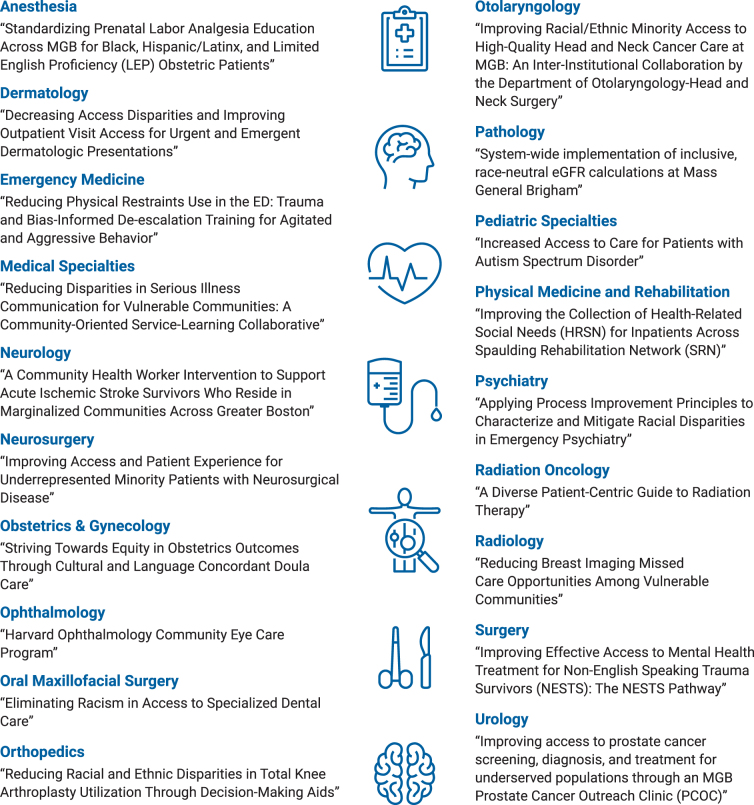
Specialty clinical equity improvement projects.

Teams from each of our 19 clinical departments, in all cases across more than one Mass General Brigham hospital, are working on health equity improvement projects to address a known disparity in their field (Fig. 3). Each specialty department identified a racial disparity and proposed a multiyear quality improvement project designed to address the gap. Teams participated in a Clinical Process Improvement Program to gain skills in defining the problem and developing an appropriately scoped solution. Post-training, selected projects were funded through grants ranging from $10,000 to $100,000 each to implement their equity-improvement solutions.

As an example, the team from our obstetrics and gynecology departments initiated a project to address racial and ethnic inequities in maternal health outcomes. Nationally, Black individuals are more likely to be delivered by cesarean and have higher rates of maternal morbidity and mortality.^23,24^ To address these issues, the team developed a program to provide racially and ethnically concordant doulas for Black and Indigenous patients in our system on the basis of their elevated risk of adverse pregnancy outcomes. The doulas provide support during pregnancy, labor, and the postpartum period.

Outcomes monitored for this initiative include patient satisfaction, rates of cesarean delivery, and breastfeeding among program participants, and early findings suggest cesarean rates lower than the national goal and lower than nonparticipating birthing individuals within our system.

The overall goal of the specialty projects is to build an organizational culture of equity improvement and to inform institutional policies to promote health and health care equity. To close equity gaps, programs are encouraged to leverage enterprise-level data and to engage subject matter and community experts to guide the intervention design and conduct, with utilization of test of change models to iterate on improvement processes.

#### Eliminating racialized medicine

Race, as a social construct and not a biological one, is not a reliable proxy for genetic difference and should not be used to determine clinical care.^25^ However, race is still inappropriately used as an input in many diagnostic algorithms and practice guidelines. The use of race in clinical decision making and risk assessment harms patients of color because it influences the type of health services and treatments offered to them. Under the *Eliminating Racialized Medicine* workstream, we are reconsidering the use of race in clinical risk calculators and predictive models within our Epic Systems build. Our goal is to quantify the scope of these instances of race-adjusted algorithms and demonstrate reduction.

We developed working groups of subject matter experts to create data-driven plans to eliminate the use of race in medical calculators while keeping them scientifically rigorous. One example is the traditional use of race in the calculation of estimated glomerular filtration rate (eGFR), a measure of renal function. This has contributed to the underdiagnosis and undertreatment of Black patients with chronic kidney disease, including consideration for transplantation when indicated. We adopted a race-neutral calculation of eGFR, which has since been recommended by the American Society of Nephrology and the National Kidney Foundation. As a result, one in three Black patients at Mass General Brigham have been reclassified to reflect the more severe chronic kidney disease stage they actually suffer.

In circumstances in which changes to race-embedded calculators lie beyond our direct control, we exert advocacy for change and/or elect to send nullified race inputs externally, as is the case for analytics related to electrocardiogram interpretation, for example.

### Leadership/employees/culture

#### Developing enterprise-wide antiracism education

We recognize that each of our 80,000 employees are at varied points along their personal journeys toward antiracism. Providing our workforce with foundational elements of health equity and workforce diversity and inclusion is central to the work of UAR. Diversity, Equity and Inclusion, and Human Resources leadership have designed, tested, and deployed two large-scale and virtual trainings for the workforce; completion of both are conditions of employment within Mass General Brigham and are assigned as part of the annual compliance cycles.

In the first, “Stepping Stones,” allegories that provide understanding of concepts such as inequity, interpersonal, internalized, and structural racism are reviewed by the learner. The concluding module incorporates these concepts into reflections into ways structure and policies within our health system may contribute to inequitable outcomes and a noninclusive workplace culture.

In the second, “Ending Racism: Everyone's Responsibility,” using real-world examples gleaned from Mass General Brigham staff as a basis, participants engage in interactive and iterative case-based learning about the ways in which daily interactions and decisions may perpetuate structural racism. Each of these trainings is accompanied by a facilitation guide available for teams that wish to debrief the trainings in group settings.

#### Instances of racism reporting

To become an antiracist organization, it will be critical to implement procedures to identify, not deny, and address in a trusted manner the instances of interpersonal racism that occur within our system. To do this, we created a multidisciplinary work group to better understand reporting preferences, availability, and barriers for our staff and patients. Patient safety, human resources, police and security, equity, compliance, and employee assistance experts were all represented in the group, which first set about to craft an enterprise-wide policy that sets expectations for behavior of our patients, their families and visitors, and our research participants.

The new enterprise “Patient Code of Conduct” spells out algorithms for management of patients who exhibit racist, discriminatory, or hostile behaviors within our care communities. For those patients with repetitive behaviors that violate the code, dismissal from Mass General Brigham care may be called for.

Beyond codifying policy, the efforts of this workstream include cataloging existing and building new and trusted reporting and response mechanisms when instances of racism and discrimination occur within our organization. The same workgroup drafted standardized algorithms for system response to reports of discrimination, whether by patients or employees. Equity-informed review of reports, appropriate investigation and reconciliation, feedback to the reporter, and logging of the report for tracking purposes are delineated and are being piloted at several sites across the enterprise.

In attempt to ensure informed and antiracist responses, we developed a synchronous facilitated virtual “Upstander” training designed for staff managers, training program directors, and others likely to be on the front lines of receiving reports, in addition to the required trainings above.

### Community health and policy advocacy

#### Community-based clinical equity programs

**FIG. 4.**
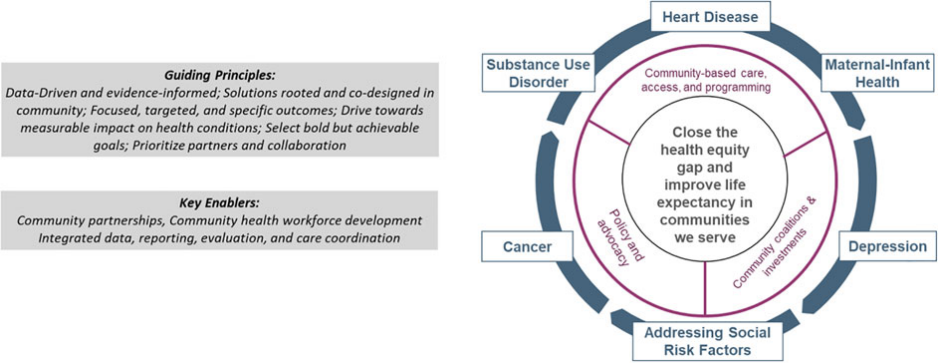
Achieving community health equity.

Structural racism has contributed to variation in clinical outcomes among the communities we serve. For example, hemoglobin A1c control is met in 73% of primary care patients with diabetes in Newton, Massachusetts, a predominantly wealthy and White Boston suburb, compared with only 58% in Revere and 60% in Chelsea, nearby cities that are both predominantly working-class communities of color. To address these disparities, we have partnered with communities to work toward removing barriers to care for patients and community members (Fig. 4).

One strategy for reaching communities with a high burden of disease and chronic conditions is to bring our care to them through mobile clinics. As part of the *Community Outcomes* workstream, we have repurposed community van service lines that visit 20 neighborhoods in the greater Boston area. On the heels of successful mobile COVID-19 programming, including the provision of COVID-19 testing, therapeutics, and vaccination, we developed a clinical service line to address the burden of cardiometabolic disease in the area through hypertension screening and treatment, and will expand to provide substance use disorder therapy in the coming months.

Parallel to the nutrition equity work we have undertaken in our clinical spaces, we partnered with community-based organizations to make nutritious food available to members of our communities with food insecurity, through delivery of food boxes, debit cards with which specific food items may be purchased, and investment in teaching kitchens among other mechanisms. Under the *Community Outcomes* workstream, we will continue to drive improved community outcomes in the neighborhoods we serve by leveraging our clinical strengths to develop community-informed evidence-based service models.

#### External policy and advocacy

Local, state, and national policies have impact on the health and well-being of our patients and communities. For example, public transportation policies affect access to employment, healthy food, and health care services. Therefore, we have a responsibility to leverage our position to influence policies that impact drivers of health. Under the *External Policy and Advocacy* workstream, we work in partnership with Mass General Brigham's Office of Government Affairs to advocate for antiracist public policies at the local, state, and national levels, recognizing external policy as an effective lever to increase social and racial justice among our patients and the communities we serve.

After a thorough review of those policy priorites that would be most impactful in the elimination of health inequities, we elevated three areas, telehealth access, opiate use disorder, and social risk mitigation, for particular policy focus.

## Conclusion

UAR provides a roadmap to implement large-scale equity interventions across an entire system. One of the most important elements of UAR is that it simultaneously addresses racism embedded in various areas of health care. We are building a culture of equity by investing in our workforce and by re-evaluating and restructuring our system-wide policies and practices to produce equity. We are expanding access to care through payer contracting, translation services, and digital health; eliminating racism in clinical care and protocols; developing new care models to address SDOH and improve community health outcomes; advocating for antiracist internal and external policies; and training our workforce to work alongside us in this initiative.

Collecting reliable data, setting clear objectives, and conducting rigorous evaluation are essential to monitor progress of our racial equity work. In addition, transparency around our processes and outcomes is vital to encourage trust and participation in the work. UAR is our commitment to making a tangible difference in health equity and eliminating the many impacts structural racism has on our employees, patients, and communities.

## Abbreviations Used

CHW: community health worker
DAC: digital access coordinator
eGFR: estimated glomerular filtration rate
LEP: limited English proficiency
REaL: race, ethnicity, and language
SDOH: social determinants of health
UAR: United Against Racism
